# Flexible metallic core–shell nanostructured electrodes for neural interfacing

**DOI:** 10.1038/s41598-024-53719-4

**Published:** 2024-02-14

**Authors:** Beatriz L. Rodilla, Ana Arché-Núñez, Sandra Ruiz-Gómez, Ana Domínguez-Bajo, Claudia Fernández-González, Clara Guillén-Colomer, Ankor González-Mayorga, Noelia Rodríguez-Díez, Julio Camarero, Rodolfo Miranda, Elisa López-Dolado, Pilar Ocón, María C. Serrano, Lucas Pérez, M. Teresa González

**Affiliations:** 1 Fundación IMDEA Nanociencia, Calle Faraday 9, 28049 Madrid, Spain; 2 Departamento de Física de Materiales, Universidad Complutense de Madrid, Plaza de las Ciencias S/N, 28040 Madrid, Spain; 3 Max Planck Institute for Chemical Physics of Solids, Dresden, Germany; 4 Instituto de Ciencia de Materiales de Madrid (ICMM), CSIC, Calle Sor Juana Inés de la Cruz 3, 28049 Madrid, Spain; 5 Present Address: Animal Molecular and Cellular Biology group (AMCB), Louvain Institute of Biomolecular Science and Technology (LIBST), Université catholique de Louvain, Place Croix du Sud 5, 1348 Louvain la Neuve, Belgium; 6 Hospital Nacional de Parapléjicos, SESCAM, Finca la Peraleda S/N, 45071 Toledo, Spain; 7 Department de Física de la Materia Condensada and Instituto “Nicolás Cabrera”, Universidad Autónoma de Madrid, 28049 Madrid, Spain; 8 Design and development of Biomaterials for Neural Regeneration, HNP-SESCAM, Associated Unit With CSIC Through ICMM, Finca La Peraleda S/N, 45071 Toledo, Spain; 9 Departamento de Química Física Aplicada, Universidad Autónoma de Madrid, 28049 Madrid, Spain

**Keywords:** Nanoscale biophysics, Nanomedicine, Biomedical engineering, Implants, Electronic properties and materials, Nanowires, Synthesis and processing

## Abstract

Electrodes with nanostructured surface have emerged as promising low-impedance neural interfaces that can avoid the charge‐injection restrictions typically associated to microelectrodes. In this work, we propose a novel approximation, based on a two-step template assisted electrodeposition technique, to obtain flexible nanostructured electrodes coated with core–shell Ni–Au vertical nanowires. These nanowires benefit from biocompatibility of the Au shell exposed to the environment and the mechanical properties of Ni that allow for nanowires longer and more homogeneous in length than their only-Au counterparts. The nanostructured electrodes show impedance values, measured by electrochemical impedance spectroscopy (EIS), at least 9 times lower than those of flat reference electrodes. This ratio is in good accordance with the increased effective surface area determined both from SEM images and cyclic voltammetry measurements, evidencing that only Au is exposed to the medium. The observed EIS profile evolution of Ni–Au electrodes over 7 days were very close to those of Au electrodes and differently from Ni ones. Finally, the morphology, viability and neuronal differentiation of rat embryonic cortical cells cultured on Ni–Au NW electrodes were found to be similar to those on control (glass) substrates and Au NW electrodes, accompanied by a lower glial cell differentiation. This positive in-vitro neural cell behavior encourages further investigation to explore the tissue responses that the implantation of these nanostructured electrodes might elicit in healthy (damaged) neural tissues in vivo, with special emphasis on eventual tissue encapsulation.

## Introduction

Neural interfaces play a fundamental role in electrophysiology, neuromodulation and neurochemical sensing, allowing a better understanding of the brain functioning and the development of new diagnostic and treatment strategies for neurological disorders^1–3^. Their electrical performance as well as their integration within the neural tissue are the most relevant challenges neural interfaces meet^4–6^. Electrodes with improved electrical properties are now in demand with special focus on decreasing the electrode impedance, for better signal recording and more efficient stimulation^3^. In particular, lower impedance interfaces allow to reduce the size of the electrodes, enabling more localized electrophysiological procedures^7,8^. This allows both to detect signals even at a single‐cell level and to more precisely stimulate specific neural populations, limiting the possible side effects derived from unspecific stimulation^5,9^.

Depositing materials with nanostructured patterns or 3D vertical nanostructures^10–14^ is one of the strategies followed to decrease the impedance of the electrodes by increasing their effective area^10,15–18^. Among the studied nanostructures, vertical free‐standing nanowire (NW) arrays emerge as promising candidates for the design of neural electrodes. Minimal impedance can be achieved by combining the high aspect ratio of NWs with high NW density^19,20^. For example, a large impedance reduction has been reported in ZnO NWs compared to bare Au substrates (12 times lower at 1 kHz)^16^, which was further reduced by coating ZnO with Au and PEDOT (35 times lower). In the work of Boehler et al.^15^ a grass‐like Pt layer is deposited by wet electrochemical processes on top of smooth Pt electrodes. They observed that the consequent increment in the effective area translated into an impedance more than 60 times lower than that of unmodified bare Pt electrodes, with even lower impedance values when increasing the nanowire length^20^. Similarly, a 10 factor decrease was obtained by Ganji et al.^21^ using polycrystalline and porous Pt nanorods of 300–400 nm in height, obtained by selective chemical dissolution of Ag from cosputtered PtAg alloys.

Furthermore, nanostructured substrates with vertical NWs have shown to improve neural cell adhesion in vitro^22,23^, which is expected to improve neural cell and tissue responses to the electrodes^24,25^. Specifically, Xie et al.^23^ showed good neuronal pinning by non‐invasive NWs of 150 nm in diameter and 1 μm in height, independently of the composition of the NWs. Higher viability was reported in HEK‐293 cells (human embryonic kidney cells) when cultured on NWs substrates compared to flat ones, suggesting that nanostructured substrates are less invasive^16^.

Finally, these nanotopographies have also the potential of tailoring cellular responses^26^. In‐vitro studies have shown that nanostructured substrates, including the use of NWs, can affect neural cell differentiation^27^, promoting astroglia reduction and supported neuronal growth over glia^28–30^. In addition, substrates with standing NWs are being studied as platforms to promote neural guidance^28,31^ and to modulate neural activity, which are of high interest in regenerative medicine and tissue engineering^26^. In all these applications, a fine control on the NW morphology and composition is of the utmost importance for obtaining optimum performance and tissue response.

Recent developments in the fabrication of NWs are moving towards the integration of several materials into a nanostructure to exploit the physicochemical properties of the different materials involved^32–35^, properties that are being explored in the field of biomedicine. For example, in the work of Hopkins et al.^36^ Ni–Au core–shell NWs were used under radio-frequency-mediated for hyperthermia treatment. Polymeric composites with embedded Ag-Au NWs has shown promise for the development of implantable soft devices that can be conformally integrated in skin and cardiac tissue for continuous electrophysiological recording and electrical and thermal stimulation^37^. Striped Au/Ag NWs have also been investigated for detection of specific nucleic acid molecules when functionalized with a silica shell of controllable thickness (6–150 nm) to protect Ag segments from oxidation^38^.

In this context, we recently developed a novel approach^39^ to create metallic core–shell NWs based on a two-step template-assisted electrodeposition process. Template-assisted electrochemical deposition is one of the most efficient techniques to grow 1D metallic nanostructures, which can be converted into core–shell nanostructures using different approaches^35,40–43^. Physical growth methods, such as sputtering and electron beam evaporation, do not generate a conformed shell over the NWs and may show adhesion issues^44,45^. On the other hand, autocatalytic chemical methods in solution, as electroless plating, have been proposed as versatile approaches to fabricate conformal coatings on previously electrodeposited NWs, producing the radial structure^36,46,47^. However, since the galvanic replacement usually dominates the growth process, it can result in hollow covers^48^. Alternatively, there is also a set of strategies in which a tube is grown first at the walls of the template nanopores and, afterwards, the core is grown by electrochemical deposition inside the tubes^43,49^. Although quite versatile, these techniques do not ensure a full encapsulation of the core, which is an essential requirement for biomedical applications. Furthermore, the effective area of the coating material is smaller than it could be, directly affecting the electric properties. Our protocol instead allows the synthesis of well-controlled shells that fully cover the surface of the NW in conformal way, increasing the exposed effective area of the coating material^39^.

In this work, we apply our approach to the fabrication of flexible neural-interface electrodes coated by a network of core–shell Ni–Au NWs in vertical configuration, whose Au shell conformally covers the NWs. Our previous work^50,51^ and that of others^52–54^ demonstrate the good biocompatibility of Au nanostructured surfaces. In addition, the Au shell will allow to take advantage of well-established Au biofunctionalization protocols^47,55^. On the other hand, the Ni core offers a good mechanical stability and robustness to the NW structure, since both the yield stress and Young modulus of Ni are higher than those of Au (Yield stress Ni: 78 MPa, Au: 29–39 MPa; Young modulus Ni: 262 GPa, Au: 115 GPa)^56,57^. Additionally, the magnetic Ni core of the Ni–Au NWs can allow the combination of electric and magnetic stimuli to the neural tissue. It has been reported that applied magnetic fields can directly affect neuronal behaviour^58^. In addition, by tailoring the magnetization of a substrate, neurite outgrowth can be guided in neurons with internalized magnetic nanoparticles^59^. Magnetically directed nanowires over substrates have been used as well to induce cell orientation^60^, and neural stimulation has been demonstrated by heating through magnetic nanoparticles targeted to plasma membrane^61^.

An exhaustive morphological and structural characterization of the core–shell Ni–Au NW electrodes is presented in this work. Cyclic voltammetry (CV) has been performed to test the coating efficiency of the Au shell, as well as the increase of the effective surface area due to the nanostructured surface of the electrodes. In addition, their impedance in an electrophysiological medium has been analyzed by electrochemical impedance spectroscopy (EIS) over a wide frequency range and over a period of 7 days to compare its behavior in Ni–Au NW electrodes to that of pure Au NWs and electrodes of flat surface. Finally, we have explored the biocompatibility of these structures with rat embryonic neural cortical cells in vitro, in view of their potential use as efficient electrodes for neural interfacing.

## Results and discussion

**Figure 1:**
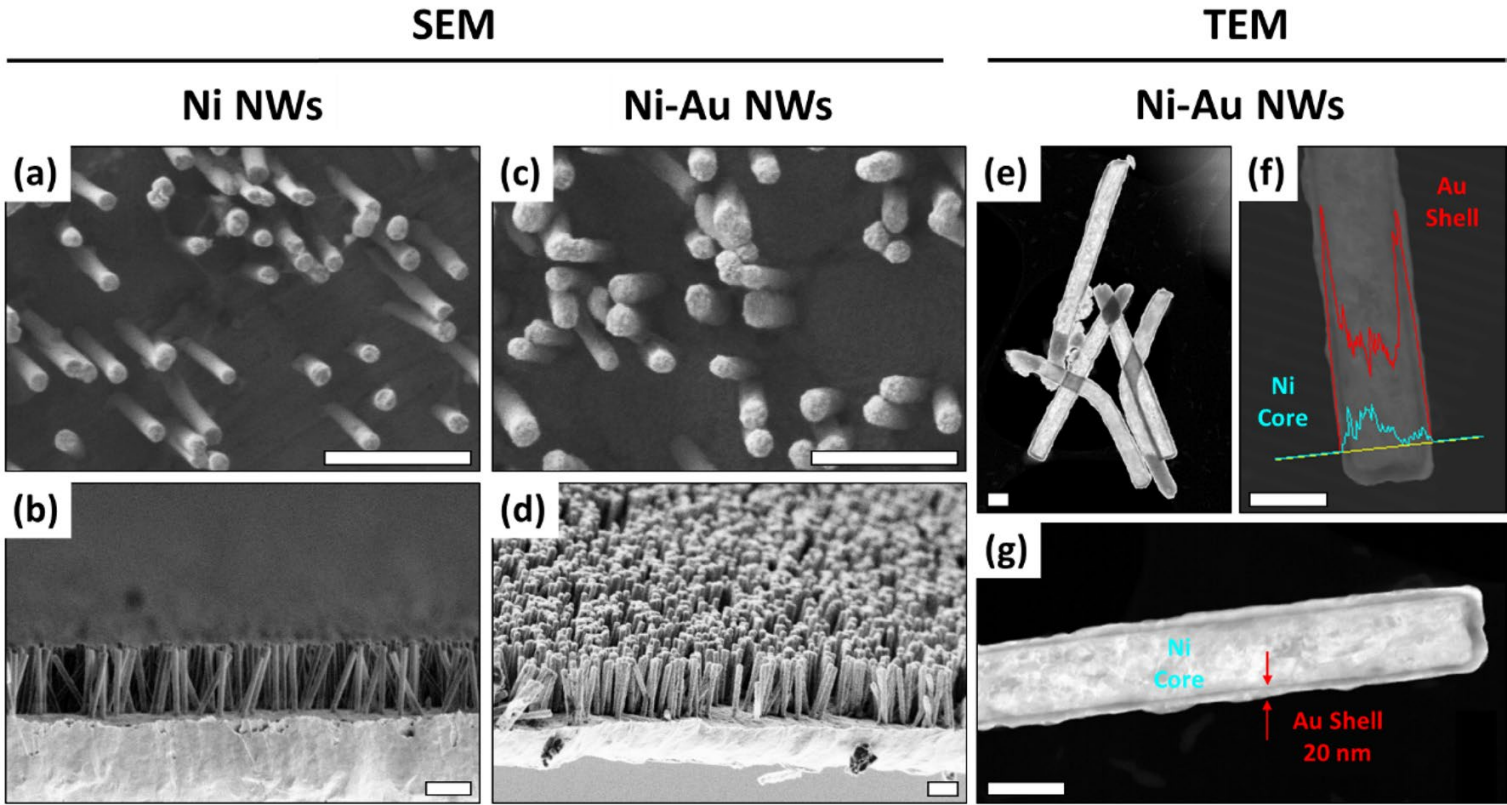
(**a**–**d**) Representative SEM images of vertical Ni (**a**, **b**) and Ni–Au core–shell (**c**, **d**) NW electrodes in top view (**a**, **c**) and cross-section (**b**, **d**). Scale bar: 1 µm in all images. (**e**–**g**) Representative TEM Z-contrast images of free-standing Ni–Au NWs detached from the electrodes with low (**e**) and high (**f**, **g**) magnification. EDX line scans along the core–shell region of the NW are superposed in the image of panel (**f**), revealing the composition of the different NW layers. Scale bars: 200 nm (**e**) and 100 nm (**f**, **g**).

Figure 1 shows SEM images of the arrays of vertical NWs on top of Au flexible films fabricated as described in the experimental section (see scheme of Fig. S1). Figure 1a,b show top and cross-section images of Ni NWs before the growth of the Au shell (Ni NW electrodes), while Fig. 1c,d show the corresponding images after the shell deposition (Ni–Au NW electrodes). In both cases, the electrodeposited NWs remain firmly attached (no fallen or absent NWs were significantly detected in any of the samples). They are randomly distributed, as expected from the pore structure of the PC template (NWs density = (6.0 ± 0.2) × 10^8^ NWs/cm^2^). The initial Ni NWs were 1.90 ± 0.03 µm in length (error bar given by the standard error of the mean value among different samples) and their averaged diameter was 136 ± 2 nm. After the Au shell deposition, a slight increase of the NW diameter is observed as expected, resulting in a 176 ± 3 nm final averaged diameter. In the images, there is an evident different morphology of the NWs before and after the growth of the Au shell, reflecting the coverage of the Ni NWs by Au.

A deeper insight into the core–shell NW structure can be extracted from TEM images. Figure 1e–g show representative images of individual core–shell NWs released from the Au base using sonication, which kept their initial length and diameter. As observed, the NWs are completely covered by Au, including the region that was attached to the Au base. This is because, during the Au base deposition over the PC templates, Au partially enters the pores giving place to an initial Au section in the NWs. The NWs have a smooth surface with a continuous Au coating. The average thickness of the shell layer determined from the TEM images is 20 ± 2 nm (see Fig. 1g). Figure 1f shows the concentration profiles for Ni and Au determined by energy dispersive X-ray spectroscopy EDX, clearly indicating a Ni core with Au as the shell of the NW.

We compared the morphology of these NW arrays to those made completely of gold (Au NW electrodes) previously reported^51^ and observed that both for Ni NWs and Ni–Au NWs the length of the NWs is significantly more homogeneous within the array than for Au NWs (see Fig. S4). In particular, the standard deviation of the NW length within a sample was more than 3 times larger for Au NWs than for Ni NWs or Ni–Au NWs (0.31 ± 0.03 µm for Au NWs to compare with 0.09 ± 0.02 µm for Ni NWs). In addition, Ni NWs could also be grown at least 15% longer than Au NWs before the grouping typical of long NWs^62^ was observed, probably because of the higher yield stress of Ni (Ni: 78 MPa, Au: 29.4–39.2 MPa)^56,57^. Therefore, Ni–Au NW electrode combine the structural advantages of the Ni core with the electrochemical and biocompatibility properties of Au.

CV in N_2_-saturated 0.5 M H_2_SO_4_ solution was performed to study the electrochemical behaviour of the Ni–Au NW electrodes. For comparison, we also characterized Ni NW electrodes, Au NW electrodes, as well as commercial Au on glass substrates (Arrandee™), which have a fully flat surface (see Fig. S4a–e).

**Figure 2:**
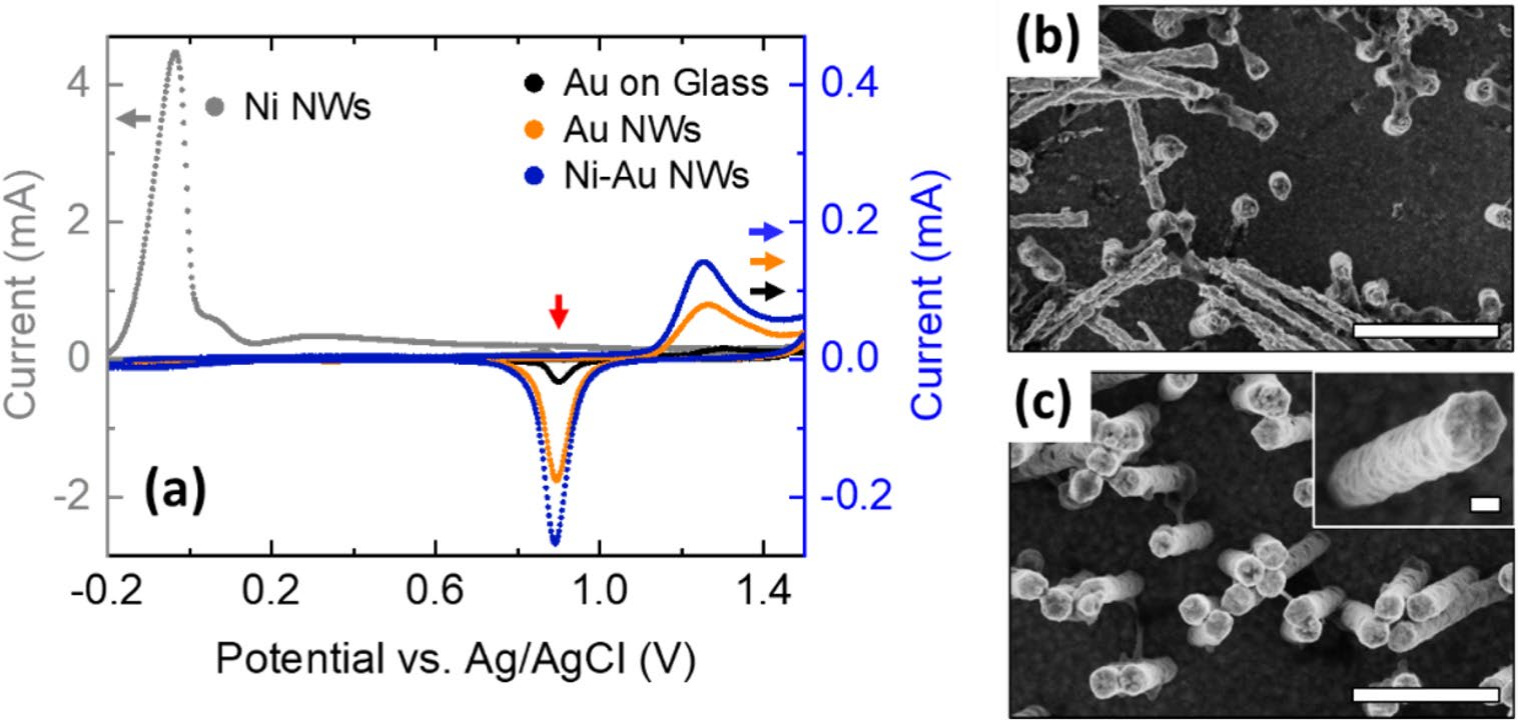
(**a**) Representative voltammograms of Au on glass substrates (black), Au NW (orange), Ni–Au NW (blue) and Ni NW (grey) electrodes. Note that the axis for Ni NWs (left axis) is 10 times larger than that of rest (right axis). Ni–Au NWs reduction peak position pointed with a red arrow. (**b**, **c**): Representative FESEM images of Ni NW electrode after 3 CV cycles (**b**) and Ni–Au NW electrode after 30 CV cycles (**c**). Scale bars: 1 µm and 100 nm (inset).

Figure 2a shows that the voltammogram of Ni–Au NW electrodes has the same profile as that of Au NW electrodes or Au on glass (more detailed in Fig. S5), with reduction peaks very close to each other (see Table 1) and in agreement within the error with the value reported in the literature^63^. In contrast, the voltammogram of Ni NW electrodes shows a high electrochemical activity with a peak at − 0.03 V, which can be attributed to Ni oxidation^64^. Importantly, this peak is not observed for Ni–Au NW electrodes, and no remarkable signs of Ni electrochemical activity can be identified in their voltammograms, confirming that, for these fabrication conditions, the Ni core of the NWs is fully covered by the Au shell. We note that, on the other hand, Ni–Au NW electrodes for which the shell deposition time was too short showed voltammograms with clear Ni activity (Fig. S6) evidencing that in those cases the Au shell coverage was incomplete.

**Table 1:** CV analysis values, and |Z| values at 1 Hz, obtained for samples with exposed Au: Au on glass substrates, Au Nw, and Ni–Au NW electrodes as well as for Au–Au NW electrodes.

	E_Au-red_ (V)	ECSA (cm^2^)	SSA (cm^2^)	ECSA/SSA	ECSA/GSA	CSC_C_ (mC/cm^2^)	|Z (1 Hz)| (kΩ)
Au on glass	0.94 ± 0.02	0.135 ± 0.008	0.13	1.04 ± 0.06	1.04 ± 0.06	0.59 ± 0.07	83 ± 25
Au NWs	0.93 ± 0.02	0.75 ± 0.02	0.71 ± 0.03	1.06 ± 0.05	5.8 ± 0.2	3.4 ± 0.2	10.8 ± 0.4
Ni–Au NWs	0.92 ± 0.02	1.09 ± 0.03	0.95 ± 0.03	1.15 ± 0.05	8.4 ± 0.2	4.6 ± 0.3	9.6 ± 0.6
Au–Au NWs	0.93 ± 0.02	0.91 ± 0.03	0.88 ± 0.04	1.03 ± 0.08	7.0 ± 0.5	4.0 ± 0.2	–

E_Au-red_: Au reduction peak voltage, ECSA: electrochemical surface area, SSA: structural surface area, GSA: geometric surface area (0.13 cm^2^ for all studied samples). SSA_Ni NW_ = 0.76 ± 0.03, CSC_C_: cathodal charge storage capacity. (Used methodology is appliable only to Au electrodes, for this reason, no values for Ni NW electrodes are listed).

Figure 2b shows a FESEM image of a Ni NW electrode after 3 CV cycles. Compared with fresh Ni NWs (see Fig. 1a), there is a significant decrease in the NW diameter which is quite inhomogeneous along the NWs, and some of the NWs are detached from the base. We attribute this degradation to the oxidation experienced by the Ni NW electrodes during the CV studies. On the contrary, no such degradation was observed for the Ni–Au NW electrodes. Figure 2c shows Ni–Au NW electrodes after 30 CV cycles in the same acidic medium. Their structure is completely preserved, with intact Au walls (see Fig. 2c inset) and no change in diameter. These results highlight the stability of the Ni–Au NW electrodes.

Table 1 summarizes the ECSA for all the studied electrodes with Au exposed, obtained from the Au reduction peak area in the voltammograms^65^. In addition to Au on glass substrates, Au NW and Ni–Au electrodes, Au–Au NW core–shell electrodes were also studied. The latter are core–shell electrodes prepared by growing an Au shell over Au NW electrodes in an equivalent way as for Ni–Au NW electrodes (see Fig. S4h,i). For all tested electrodes, the macroscopic geometric surface area (GSA) was 0.13 cm^2^. The structural surface area (SSA), considering the electrode nanostructure, was estimated from the structural parameters of the electrodes, obtained from SEM images.

As shown in the table, the ECSA and GSA for Au on glass are very similar, with an ECSA/GSA of 1.04 ± 0.06, reflecting the smooth flat surface of these substrates. In contrast, the NW structure significantly increases the active surface of the electrodes. For Ni–Au NW electrodes, the ECSA was 8.4 ± 0.2 times larger than their GSA, and more specifically ECSA Ni–Au_NW_/ECSA_Au-on-glass_ was 8.1 ± 0.5.

For Ni–Au NW electrodes, the ECSA was also 1.45 ± 0.06 times larger than that of Au NW electrodes, reflecting the area increment due to the shell thickness and the larger length of the core Ni NWs (1.90 ± 0.03 µm in comparison with that of the Au NWs 1.73 ± 0.08 µm). Due to the difference in the homogeneity of the NW length in both samples described above, to evaluate with better certainty the contribution of only the Au shell to the ECSA of the electrodes, we decided to compare the ECSA of Au NW electrodes with that of Au–Au NW core–shell electrodes, simply prepared coating the Au NWs with an additional Au shell. The ratio between the ECSA of Au–Au NW electrodes and Au NW ones is 1.21 ± 0.05, which agrees with the expected value from their calculated SSA (SSA_Au–Au NW_/SSA_Au NW_ = 1.24 ± 0.08). This result serves as confirmation that the Au shell thickness in the NWs is 20 nm, as estimated from the TEM images.

Finally, Table 1 displays the available cathodal charge storage capacity (CSC_C_) extracted from the recorded voltammograms (at v = 50 mV s^−1^ in 0.5 M H_2_SO_4_ aqueous solution, see Methods section). Ni–Au NW electrodes have a CSC_C_ 7.8 ± 0.4 times larger than the Au on glass substrates and 1.4 ± 0.1 times larger than the Au NW electrodes, showing a reasonable concordance with the ECSA previous results, and reflecting the effect of the nanostructure and the potential of the Ni–Au NW electrodes with improved electrical properties respect to planar references.

**Figure 3:**
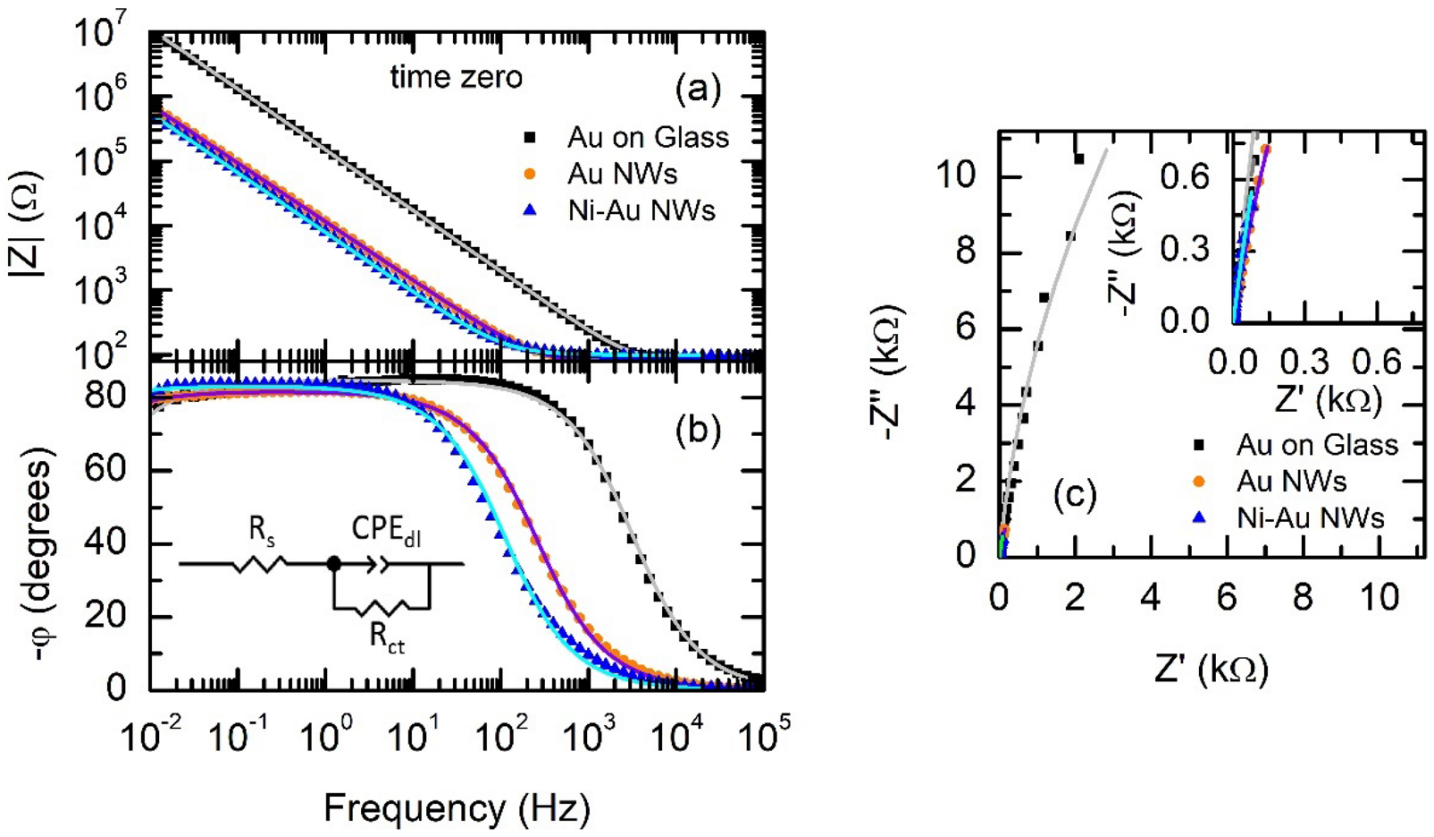
Representative EIS measurements at time zero of Au on glass substrates (black), Au NW (orange), and Ni–Au NW (blue) electrodes. Bode Plots represent the impedance module (|Z|) vs frequency (**a**) and phase (φ) vs frequency (**c**). Nyquist Plot represents imaginary (Z’’) vs real (Z’) impedance. Continuous lines over the experimental points are fits to data of the model shown in the inset of part (**b**) (see text for details).

To evaluate the change in impedance resulting from the nanostructure of the electrodes, we performed EIS measurements at room temperature in phosphate buffered saline (PBS), a physiological-type solution^15,66^. Figure 3 shows a summary of the Bode and Nyquist plots for Ni–Au NW electrodes in comparison with those of Au on glass surfaces and Au NW electrodes, obtained 10 min after immersing the samples in the solution (time zero). The figure shows representative curves of each electrode type. At least 4 samples of each type were studied, obtaining the same EIS profiles with small variations in |Z| for the prepared electrodes, with values at 1 Hz given in Table 1.

**Figure 4:**
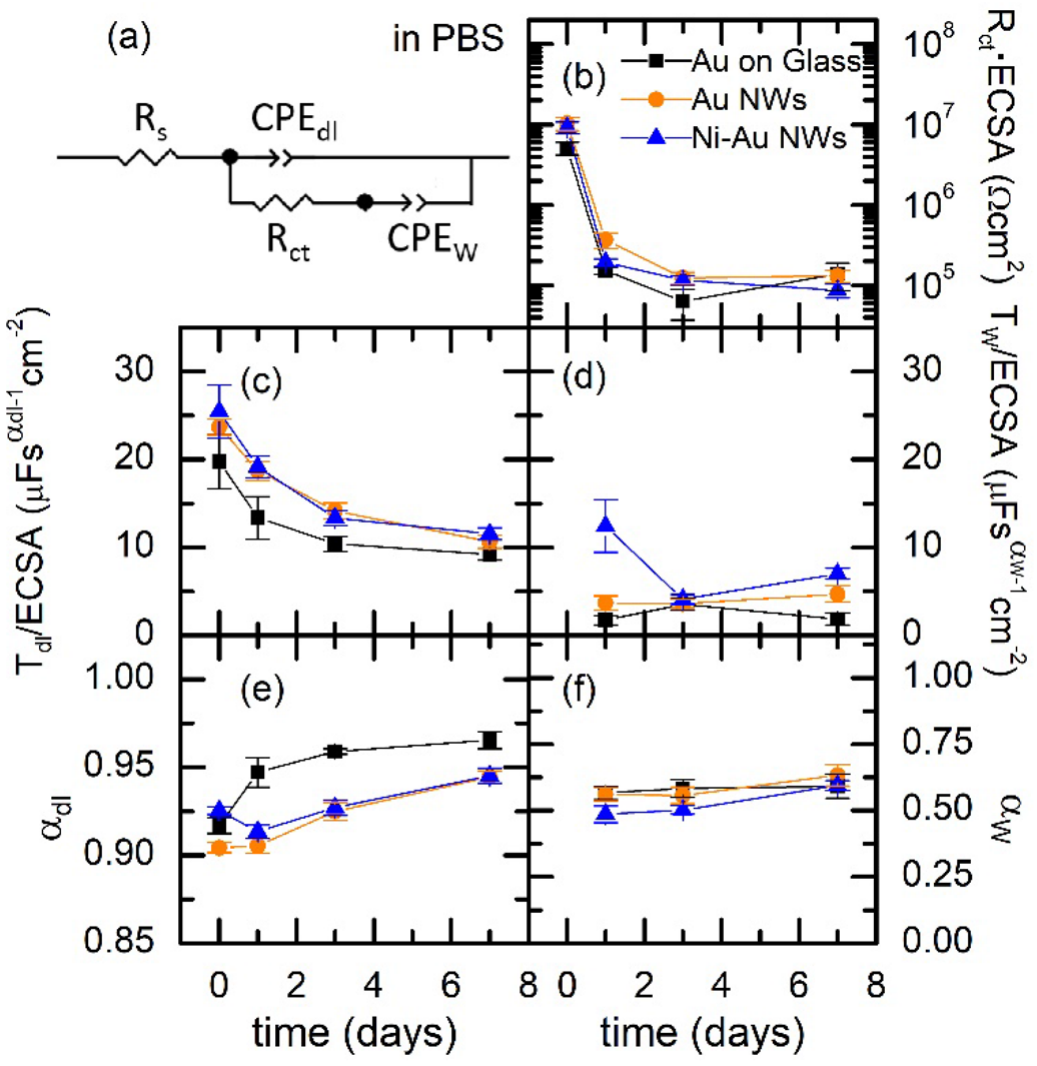
(**a**) Equivalent electrical circuit used to model the impedance of the studied samples after 1 day or more in PBS. Z_CPE__dl_ = 1/(T_dl_·(j·ω)^α^_dl_) and Z_CPE__w_ = 1/(T_W_·(j·ω)^α^_w_). (**b**)–(**f**) averages over 3–4 samples of the parameters resulting from fitting the impedance expression of the circuit of part (**a**) to the Bode and Nyquist plots of Au on glass (black), Au NW electrodes (orange) and Ni–Au NW electrodes (blue) obtained over a period of 7 days. Note that, the model of Fig. 3b inset, without CPE_w_, was used at time zero.

Solid lines in Fig. 3 correspond to the resulting curves of fitting the electrical equivalent circuit of Fig. 3b inset to the experimental data. In the model, Z_CPE__dl_ = 1/(T_dl_·(j·ω)^α^_dl_) (see also the electrode characterization subsection in experimental methods below)^67,68^. High values for charge transfer resistance R_ct_ were obtained (values a time zero in Fig. 4b), which points to a quasi-ideal polarizable behaviour at time zero. At high frequencies (above ≈ 10^3^ Hz), the impedance is independent of the frequency, so the |*Z*| profile is almost flat for all the electrodes (Fig. 3a), while the phase angle (φ) is almost zero (Fig. 3b). The impedance in this frequency range can correlate with the solution resistance (R_S_), with variations between 60 and 105 Ω in our experiments.

At frequencies lower than 10^−3^ Hz, log |*Z*| in Bode plots follows a linear dependence with the log frequency. This behaviour is ascribed to the capacitive behaviour of the electrode/electrolyte interface (double layer charging). In this frequency range, the impedance module of the nanostructured electrodes is almost 1 order of magnitude lower than that of the flat Au on glass surfaces (see the values at 1 Hz in Table 1), correlating with the obtained ECSA values. In fact, we observed a very good scaling of the whole |Z| vs frequency plots with the obtained ECSA values (see Fig. S7). In addition, the impedance of the Ni–Au NW electrode has the same profile as that of the Au NW electrodes, with slightly lower impedance values, which differs from that of the Ni NW electrodes shown in Fig. S8, especially in the low frequency range.

In order to explore the stability of the NW structure, we studied the EIS evolution over a period of 7 days for all samples. Although the usual range for neural applications is between 1 and 1000 Hz^69,70^, we have chosen to include the significantly less studied range 0.01–1 Hz to get a full picture of processes at the electrode/electrolyte interface, and identify the contribution of direct charge transfer and diffusion. Figure 4b–f summarizes the time evolution of the averaged fitting parameters (examples of the time evolution of the Bode and Nyquist plots for individual samples of each type are shown in Fig. S9, at least 3 different samples of each kind were studied). R_S_ is not included in Fig. 4 as it is a property of the electrolyte as mentioned above.

While, at time zero, we used the circuit model of Fig. 3b inset, after 24 h, the Bode and Nyquist plots evolved from those of Fig. 3, and this simple circuit was not enough to obtain a good fit at low frequencies. We used instead the electrical equivalent circuit of Fig. 4a, where a diffusion term Z_CPE__W_ = 1/(T_W_·(j·ω)^α^_W_) was added (see also the electrode characterization subsection in experimental methods below)^67,68^. In Fig. 4c, during the first 3 days, T_dl_ decreases with the same trend for the three samples. This evolution cannot be simply understood as a decrease in the capacitance of the electrodes as T_dl_ is intrinsically coupled to α_dl_, and we observed that the decrease of T_dl_ correlated with an increase in α_dl_, i.e., with a more capacitive character of the CPE_dl_ element. In any case, the α_dl_ values are in all cases very close to 1, meaning that capacitive charge transfer through the double layer dominates over faradaic charge transfer in these electrodes, the former being a less detrimental mechanism for the electrode and the neural tissue and therefore more favourable for neural interfacing applications. After 3 days, the values of all parameters remained fairly stable up to 7 days, with occasional increase of the diffusion impedance component in some of the tested samples which could be due to the deterioration of their electrode base among other causes. The parameter α_w_ was left as a free parameter and observed to converge to a value very close to 0.55. While a pure Warburg model predicts a value of α_W_ = 0.5, a deviation of this value can reflect a non-uniform or multiple-path diffusion^71^. In addition, a value of 0.55 has been described to reproduce the diffusion in electrodes with disc shape, as the present case ^68^. In particular, the average value of α_W_ after 24 h was 0.58 ± 0.04 for Au on glass, 0.58 ± 0.03 for Au NW electrodes and 0.53 ± 0.05 for Ni–Au NW electrodes. Both T_w_ and α_w_ remained fairly stable in the studied time period for the three samples. In contrast, for Ni NW electrodes, (see Fig. S10) α_w_ starts at 0.5 and acquires a value approaching 0.7 after 3 days, deviating from the Warburg diffusion model described as a semi-infinite diffusion. Ni NW electrodes show an electrochemical activity, which is supressed in the Ni–Au NW electrodes that behave instead as only Au NW electrodes.

In Fig. 4, the resulting R and T parameters are normalised by the obtained ECSA values (see also Fig. S11). In this figure, it is important to note that the values of T_dl_ and T_w_ for the different samples do not have the same units, as those depend on α_w_ and α_w_, respectively. These do not have identical values, as shown in Fig. 4e,f, but they are very similar. The differences between values for different types of samples are below 4% for α_dl_ and less than 15% for α_W_. Taking into account this consideration, Fig. 4c shows a rather good agreement for the T_dl_/ECSA values of the three samples. This is the parameter that dominates de impedance above 1 Hz and accounts for the good scaling of the overall |*Z*| curves with the ECSA (Fig. S7). We observe therefore that the ratio between the double-layer impedance of the electrodes, determined by their different ECSA, is preserved in the 7-days studied period, highlighting the stability of the Ni–Au NW electrodes and their similarity to Au NW electrodes.

**Figure 5:**
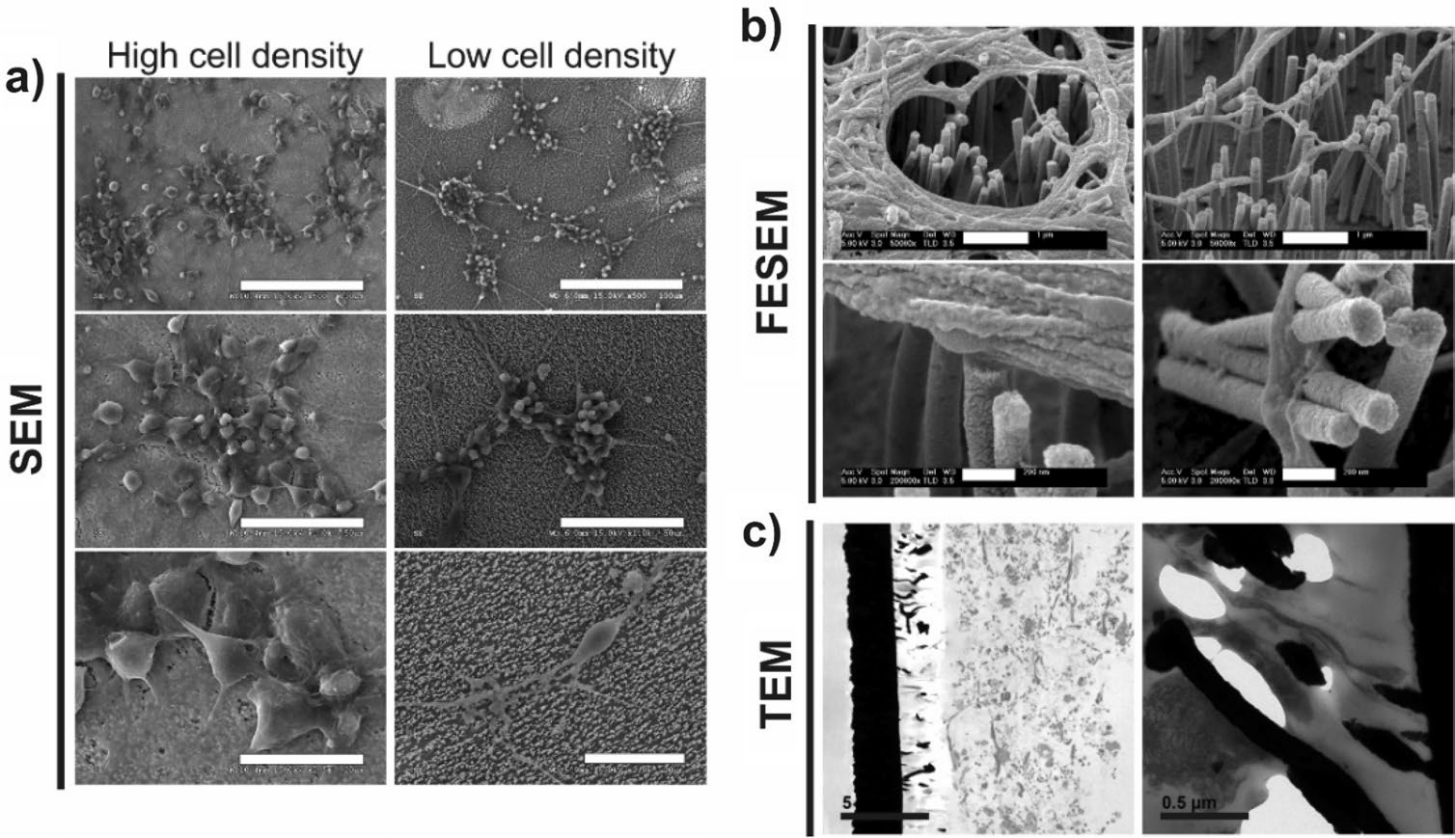
Morphological evaluation of rat neural cortical cell cultures at high- and low-density seeding conditions on Ni–Au core–shell NW electrodes. Representative SEM (**a**), FESEM (**b**) and TEM (**c**) micrographs of cultures at 14 days are shown. Scale bars in SEM images represent 100 µm (top), 50 µm (middle) and 20 µm (bottom); in FESEM, 1 µm (top) and 200 nm (bottom); and in TEM, 5 µm (left) and 0.5 µm (right).

We finally assessed the biocompatibility in vitro of these core–shell nanostructured electrodes with neural cells derived from progenitor cells isolated from the cerebral cortices of rat embryos (ENPCs). As demonstrated by SEM, ENPCs properly attached and well spread on top of PLL-coated Ni–Au NWs after 14 days when cultured at both favourable high-density conditions (75,000 cells cm^−2^) (Fig. 5a, left column) and a lower cell seeding density (25,000 cells cm^−2^) (Fig. 5a, right column). In both cases, cells displayed a typical neural morphology, with capacity to form interconnected cultures as in control (glass) substrates. Detailed observation by FESEM revealed a close contact of cells components, both somata and neurites, with the NWs (Fig. 5b). Cells with either apparent perforation of plasmatic membranes or a NW-pierced morphology were not found. High-magnification studies by TEM further corroborated these findings and demonstrated a close contact of neural cells with both the active part and the base of Ni–Au core–shell NW electrodes (Fig. 5c).

**Figure 6:**
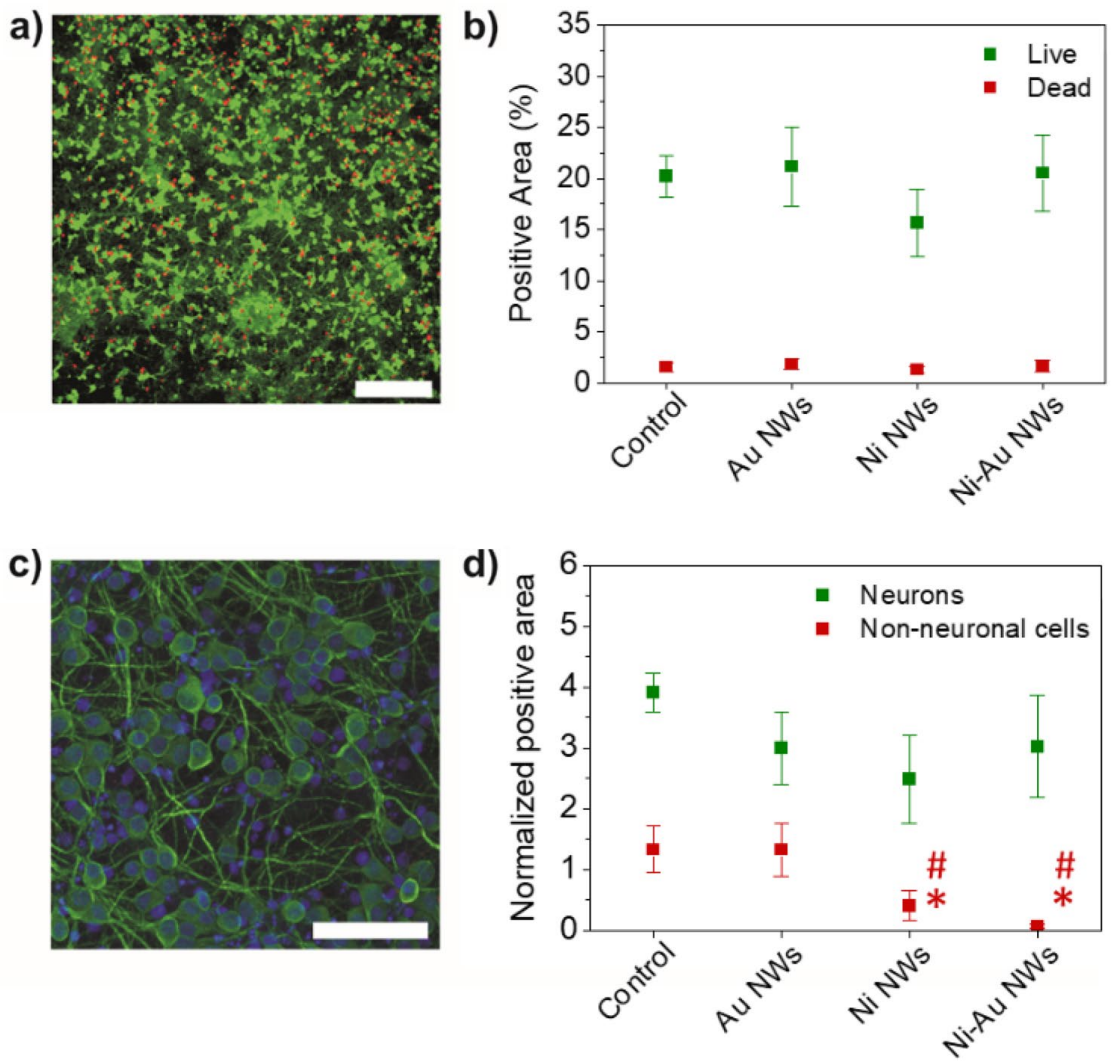
Viability (**a**, **b**) and differentiation (**c**, **d**) studies of rat neural cortical cells cultured on Ni–Au NWs at 14 days by confocal laser microscopy. (**a**) Representative images of the cultures with alive cells labelled in green (calcein) and dead cells in red (EthD-1). (**b**) Normalized positive area for alive and dead cells. (**c**) Representative images of cultures with neurons labelled for MAP-2 (green) and non-neuronal cells including glial cells for vimentin (red). Cell nuclei were stained with DAPI (blue). (**d**) Normalized positive area for neurons (MAP-2^+^) and non-neuronal cells (vimentin^+^). Values for glass coverslips (control) and Au NW and Ni NW electrodes are included as a reference (extracted from ^51^) Statistics: *p < 0.05 with respect to control glass substrates and ^#^p < 0.05 with respect to Au-NWs. Scale bars represent 150 µm in both images.

Cell viability of ENPC cultures on Ni–Au nanostructured electrodes was then evaluated and compared with previous published results of these same cells with Au and Ni NW electrodes^51^ (Fig. 6a,b). Importantly, a majority of the electrode surface was covered by live cells in a comparable manner to those on control (glass) samples (p = 0.953 for the area of live cells and p = 0.862 for the area of dead cells). Interestingly, the appreciable but not significant decrease in cell viability previously reported for Ni NWs electrodes was reverted by the presence of the Au shell in Ni–Au NW electrodes, approaching values of control and Au NW electrodes. Cell differentiation to either neuronal or non-neuronal phenotypes was investigated by immunofluorescence studies of MAP-2 (neuronal cytoskeleton protein) and vimentin (non-neuronal cytoskeleton protein) (Fig. 6c,d). Interestingly, neurons were predominant on Ni–Au NWs, as on control substrates and Au NW electrodes, and expected for these cells under these culture conditions (p = 0.358). As for viability results, we again observed a closer behaviour to Au NW electrodes rather than to Ni NW ones, in which the area covered by neurons was noticeable, but not significantly, decreased. On the contrary, the amount of non-neuronal phenotypes including glial cells was significantly diminished on these core–shell nanostructured electrodes with respect to control (glass) substrates (p = 0.024) and Au NWs (p = 0.021), but not in comparison to Ni NWs. Similar reduction was previously reported for Ni NW electrodes with respect to control (p = 0.035) and Au NW electrodes (p = 0.032)^51^. These findings seem to prove that, even when the Au shell covering the Ni core was capable of protecting neural cell viability and neuronal differentiation, glial cells were impacted. As flat Ni electrodes did not generate such effect^51^, the observed glial reduction for Ni NWs is likely due the NW shape mechanical properties, as for Ni–Au NWs, and not a response to the chemistry of the Ni material. This result is in agreement with previous findings in the literature that show a decrease on astrocytes presence while maintaining high neuronal coverage when nanostructures were used^30,72^. Interestingly, these nanostructured features could also impact microglia responses when implanted, as described by Nichols and colleagues with nanoporous Au surfaces^73^. In such work, authors described a significant decrease in BV-2 microglia proliferation, while morphology, viability and activation appeared unaltered with respect to control substrates. Based on these findings, the nanostructure of our electrodes may enhance neuron–electrode coupling, thus benefitting their performance.

As mentioned in the introduction, core–shell nanostructures have been used before in biological applications to take advantage of the interesting properties of the core while preserving the biocompatibility of the shell^36–38^. In this work, the core–shell architecture prevented toxicity issues previously described for Ni-based materials^74–77^. The biocompatible coating with Au of the complete surface of the Ni core avoided cells to directly interact with Ni while taking advances of its physicochemical properties for the design of the nanostructured arrays. The ability of these core–shell NWs to favour neuronal phenotypes over non-neuronal ones, including glial cells, shows great promise for their use as implantable neural interfaces in which exacerbated fibroglial responses must be minimized. Nonetheless, as for any 3D material, tissue responses including inflammation and eventual tissue encapsulation should be carefully investigated when implanted in vivo. To date, these encapsulation reactions have represented one of the major drawbacks for most of the electrodes designed and tested for recording and/or stimulation in vivo, especially relevant for their long-term performance. To this regard, flexible electrodes have robustly probed a superior capacity to prevent tissue encapsulation than rigid ones, as mechanical mismatch is known to induce chronic local inflammation of the tissues including the brain^78^. For instance, ultraflexibility and strechability seemed critical to reduce encapsulation thickness on microneedle arrays for peripheral nerves^79^. Moreover, the use of elastomeric materials with conductive properties is being also explored in the context of neural tissues to diminish these adverse encapsulation reactions^80^.

## Conclusions

The synthesis strategy proposed in this paper, which combines template-assisted electrodeposition with pulsed electrodeposition, is an efficient method to grow core–shell metallic NWs allowing an accurate control over their morphological and chemical characteristics. Longer and more homogeneous in length NWs are obtained for Ni–Au core–shell NW electrodes, with an electrochemical active area 45% larger than for pure gold NW electrodes. CV and EIS results confirmed that the Au shell fully covers the Ni core preventing any contact of the latter with the environment. The stability of the core–shell structure was further confirmed through EIS experiments up to 7 days. Biocompatibility studies in vitro with ENPCs from rat embryos revealed high viability and neuronal differentiation, accompanied with a significant reduction in non-neuronal differentiation. The eventual interest of these positive findings in vitro should be further corroborated in animal models in vivo, including sensing and stimulation experiments.

The here described core–shell protocol can be optimized to obtain shells of different materials, making possible to obtain different structures and even multishell NWs, which could be of interests in other various applications.

## Methods

All methods were performed in accordance with the relevant guidelines and regulations.

### Materials

Nanoporous templates (Whatman) were bought to Sigma-Aldrich. Chemical and biological reagents were purchased from Sigma-Aldrich and Panreac and used as received, unless otherwise indicated. Gold targets for sputtering were purchased to Leica. Platinum mesh and wire for the counter electrode was purchased from Goodfellow. Ag/AgCl reference electrodes were obtained from BASI. Antibodies were bought from Sigma-Aldrich and Invitrogen. Cell media and B-27 supplement were indistinctively purchased from Invitrogen and Thermo Fisher. All additional cell supplements were acquired from Lonza. In this study, all materials and biological samples were manipulated according to standard regulations, so no safety concerns arise.

### Fabrication of the core–shell Ni–Au nanostructured electrodes

Nanostructured electrodes were prepared by template-assisted electrochemical deposition similarly as previously described^39,51^. We used polycarbonate nanoporous membranes as templates, with 100 nm pore diameter. In these templates, nanopores are randomly distributed with a pore density of approximately 6 × 10^8^ pore/cm^2^. Electrodeposition was carried out in a three-electrode electrochemical cell using a Pt mesh as a counter electrode and an Ag/AgCl (3 M NaCl) electrode as a reference electrode. All cited potentials are referred to the standard potential of this electrode. All electrochemical processes were controlled with a Metrohm Autolab PGSTAT204 potentiostat equipped with a FRA32 impedance module. Ni was electrodeposited using a Watts-type electrolyte composed by NiSO_4_ (0.8 M), NiCl_2_ (0.2 M) and H_3_BO_3_ (0.4 M) at 45 °C. Au electrodeposition was carried out using an Orosene commercial electrolyte (ORE + 4, Italogalvano) at room temperature.

The synthesis of the Ni–Au core–shell NWs is illustrated in Fig. S1: starting from a nanoporous template (step 1), an Au layer (100 nm-thick) was sputtered on one of its sides (step 2) using a Leica EM ACE600 sputtering. This Au layer served as working electrode in the following steps. First, additional Au was uniformly deposited over the initial layer to thicken it from 100 nm to 1 µm (step 3). This was done by pulse-plating electrodeposition^51^, in order to release the stress of the layer during its deposition^81,82^. This Au film ultimately constituted the flexible supporting base of the nanostructured electrodes.

Next, the nanoporous template was filled with Ni using potentiostatic deposition at a constant potential of − 1 V (step 4). An electrodeposition time of 90 s was set to produce approximately 2 µm long Ni NWs. The PC template was then dissolved (step 5) in dichloromethane, followed by extensive and consecutive washes in acetone, ethanol and deionized water, leaving a network of vertical Ni NWs attached to the Au base.

This Ni NW electrode was immediately immersed in OROSENE, and a final Au shell was grown over the Ni NWs, again using pulse-plating electrodeposition to promote a conformed coverage (step 6). On/rest pulses of 1.5 V/0 and 0.1 s/1 s were applied (see Figs. S2b,c and S3) to obtain a 20 nm-thick Au shell.

### Electrode characterization

The morphology of the arrays of metallic NWs was studied by scanning electron microscopy (SEM) using a ZEISS EVO HD15 and a JSM 6335F microscopes. The latter was also used to measure composition via Energy Dispersive X-ray spectroscopy (EDX). Transmission electron microscopy (TEM) was used to obtain high resolution images by using a JEM 3000F microscope.

CV and EIS measurements were performed at room temperature using the same electrochemical cell and electronics described for the electrode fabrication. At least four different samples with a fixed 0.13 cm^2^ exposed area were measured per each type of electrode. CV was performed in N_2_-saturated 0.5 M H_2_SO_4_ aqueous solution, 10 min after immersing the samples in the solution, favouring their complete wetting, and ensuring the full stabilization of the three-cell system (open circuit potential value stable). Voltage was swept cyclically from − 0.2 to 1.5 V (vs. Ag/AgCl) at a scan rate of 50 mV/s. The Au reduction peak area, in the resulting voltammograms, was used to estimate the electroactive surface area (ECSA) of the electrodes via oxygen adsorption from solution method^65,83^, applying the conversion factor of 390 ± 10 µC/cm^2^ ^65^. EIS was performed in a solution of phosphate buffered saline (PBS) waiting 10 min after immersing the samples in the solution as in the case of CV (time zero measurement). EIS measurements were posteriorly repeated after 24, 72 h and 7 days, maintaining the samples immersed in the PBS solution, at room temperature and air conditions. Measurements were made in open circuit potential at 0.010 V_rms_ voltage modulation, in a frequency range from 10^5^ to 0.01 Hz. Data analysis was performed by a complex nonlinear least squares fit of the equivalent circuit models of Figs. 3b and 4a, using the software ZView (Scribner, North Carolina, USA). In the model, CPE_dl_ is a constant phase element that accounts for a non-purely capacitive double-layer capacitance with some frequency dispersion, while CPE_W_ accounts for the capacitive part of the diffusion or mass transfer component. The impedance of these terms is Z_CPE_ = 1/(T·(j·ω)^α^), where j is the imaginary number, ω = 2π·*f* is the angular frequency, and α is a dimensionless shift phase parameter with values between 1 and 0, so that when α = 1 the CPE is a pure capacitor with capacitance T, when α = 0, the CPE is a resistor with resistance 1/T and when α = 0.5 a pure Warburg diffusion element. In addition, R_S_ is the solution resistance described above, and R_ct_ = R_F_ + R_W_ is the charge transfer resistance, which includes the resistance to faradaic charge transfer (R_F_) at the electrode/electrolyte interface, and in the model of Fig. 4a, also the resistive term of the diffusion impedance (Warburg impedance Z_W_ = R_W_ + Z_CPE-W_) ^68,84,85^.

### Neural progenitor cells isolation and culture

In order to assure complete submersion and prevent floating, both flat and nanostructured electrodes were glued on glass coverslips by using medical grade silicone. Then, they were sterilized by UV radiation for 30 min in a biosafety cabinet and finally functionalized with poly-l-lysine (PLL) for 1 h (30–70 kDa; 45 μg ml^−1^), followed by a careful rinse in borate buffer (0.1 M). Neural progenitor cells were isolated from cerebral cortices of E16-E17 Wistar rat embryos as previously described^86^. All the experimental protocols for cell collection adhered to the regulations of the European Commission (directives 2010/63/EU and 86/609/EEC) and the Spanish government (RD53/2013 and ECC/566/2015) for the protection of animals used for scientific purposes. These procedures received approval from the corresponding authorities in Spain (Animal Research and Well-Being Committee and Habilitated Organ from the Hospital Nacional de Parapléjicos and Dirección General de Agricultura y Ganadería, Consejería de Agricultura, Medio Ambiente y Desarrollo Rural, Castilla-La Mancha; reference numbers 14-OH/2016 and 22-2016, respectively), and ARRIVE guidelines were taken into consideration and followed when applicable. Adult female Wistar rats were provided by a commercial supplier (Harlan Ibérica, Spain) and sacrificed at 16–17 days of gestation. A total of 5 independent cell cultures with a minimum of 3 replicates per condition in each culture were carried out (N = 15). Viability in all isolation procedures was always superior to 90%. For high-density seeding conditions, a total of 7.5 × 10^4^ cells (contained in 20–50 μl) was seeded on the top of each array and allowed to attach for 10 min. Immediately after, samples were immersed in 500 μl of complete Neurobasal™ media containing: B-27 supplement (2%), streptomycin (100 UI ml^−1^), penicillin (100 UI ml^−1^), and l-glutamine (1 mM). For low-density seeding conditions, cells were seeded at 2.5 × 10^4^ cells cm^−2^. Cultures were maintained for up to 2 weeks in a sterile incubator at 37 °C in a CO_2_ atmosphere (5%). Culture media were half replaced every 3–4 days. Cell culture progression in control samples was monitored by using an Axiovert CFL-40 optical microscope with a coupled Axiocam ICC-1 digital camera (Zeiss).

### Morphological studies of neural cells cultured on nanostructured electrodes

Cell culture samples were first washed with phosphate buffer saline (PBS) twice and fixed with glutaraldehyde (2.5% in PBS) for 45 min. After washing with distilled water, dehydration was performed by using series of ethanol solutions (30%, 50%, 70%, and 90%) for 15 min (2 washes) and a final dehydration in absolute ethanol for 30 min. Samples were then allowed drying at room temperature for at least 24 h. After mounting in stubs and coating with chromium under vacuum, the morphology of the samples was characterized by using a Hitachi S-3000N electron microscope and a field-emission Philips XL30 S-FEG microscope.

Alternatively, cell culture samples dedicated to TEM studies were fixed with a mixture of paraformaldehyde 4% and glutaraldehyde 1% in phosphate buffer for 1 h and then post-fixed in osmic tetroxide (1% in distilled water) for 1 h. Dehydration was then carried out by immersion in successive solutions of ethanol at increasing concentrations (30, 50, 70, 95, and 100%), with a final step in pure acetone. Samples were included in the resin Durcupán by consecutive immersion steps at increasing concentrations (1:3, 1:1, 3:1 in acetone). The so prepared samples were then polymerized at 60 °C for 48 h. Ultrathin sections (ca. 60 nm) were obtained and subsequently stained with uracil acetate and lead citrate. Samples visualization was carried out by using a Jeol JEM 1010 microscope (Japan) at 80 kV with a coupled camera (Gatan SC200, USA) for image acquisition.

### Viability studies of neural cells cultured on nanostructured electrodes

Cells cultures on the different substrates were first analyzed by using a Live/Dead^®^ Viability kit following the manufacturer’s instructions. This test is based on the use of calcein and ethidium homodimer-1 (EthD-1). Calcein, a non-fluorescent cell-permeable dye, gets converted into a strongly green-light emitting compound when in contact with intracellular esterases and so retained inside cells that are alive. On the contrary, EthD-1, a DNA-intercalating agent, penetrates cell membranes in dead cells and emits orange/red fluorescence after inserting into the DNA double helix. Labelled samples were visualized by using a Leica SP5 CLSM. The fluorescence of both probes was excited by using an Argon laser tuned to 488 nm. After excitation, emitted fluorescence was separated by using a triple dicroic filter 488/561/633 and measured at 505–570 nm for green fluorescence (calcein) and 630–750 nm for red fluorescence (EthD-1). Physical reflection from the metallic electrodes (non-transparent) after excitation at 488 nm was used to visualize the material structure and the relative cellular location. For quantification purposes, at least 5 images were randomly acquired for each substrate from at least 3 independent experiments and the area of live (green) and dead (red) cells measured and expressed as a percentage of the total image area.

### Differentiation studies of neural cells cultured on nanostructured electrodes

Cell cultures on the different substrates were fixed with paraformaldehyde (4% in PBS) for 15 min at room temperature and then incubated with the following primary antibodies: (1) MAP-2 for mature neurons and (2) vimentin for non-neuronal cells including glial cells. The secondary antibodies used were: Alexa Fluor® 488 anti-mouse in goat and Alexa Fluor® 594 anti-rabbit in goat (Life technologies). Both primary and secondary antibodies were prepared in PBS containing saponin (0.25%) and fetal goat serum (2%) for cell permeabilization and non-specific binding blocking, respectively. Each antibody was incubated for 1 h at room temperature in darkness. Cell nuclei were labelled with 4ʹ,6-diamidino-2-phenylindole (DAPI, 3 μM, 5 min). After immunostaining, samples were visualized by using a Leica TCS SP5 microscope. The different fluorochromes was excited and measured as follows: Alexa Fluor^®^ 488 excitation at 488 nm with an argon laser and detection in the range 507–576 nm, Alexa Fluor^®^ 594 excitation at 594 nm with a helium–neon laser and detection in the range 625–689 nm and DAPI excitation at 405 nm with a diode UV laser and detection in the range 423–476 nm. Capture conditions were stablished with appropriate positive and negative controls and maintained during the acquisition of all the images. For quantification purposes of either neuronal (MAP-2) or non-neuronal (vimentin) phenotypes, at least 5 images were randomly acquired for each substrate from at least 3 independent experiments and the respective areas obtained measured and expressed as a percentage of the total image area. Reflection mode images were again taken to observe the metallic electrodes surface and respective cell location in all cases.

### Statistics

Parameters were expressed as the mean ± standard error of the mean (in all cases, n ≥ 3). When necessary, statistical analyses were performed by using IBM SPSS Statistics software (version 28.0.1.0). Comparisons between two groups were carried out by using the T test. In all cases, the significance level was defined as p < 0.05.

### Supplementary Information

Supplementary Information.

## Data Availability

The data used in this work are available upon reasonable request from the corresponding author.
