# Supplementary material for: Flexible metallic core–shell nanostructured electrodes for neural interfacing
Source: Sci Rep. 2024 Feb 14;14:3729. doi: 10.1038/s41598-024-53719-4 (PMC10866994; doi:10.1038/s41598-024-53719-4)
Supplement: Supplementary file 1 — Supplementary Information. [file 41598_2024_53719_MOESM1_ESM.docx]

Supporting Information

**Flexible metallic core-shell nanostructured electrodes for neural interfacing**

Beatriz L. Rodilla^a,b, ‡^, Ana Arché-Nuñez^a, ‡^, Sandra Ruiz-Gómez ^c^, Ana Domínguez-Bajo^d‖^, Claudia Fernández-González^a^, Clara Guillén-Colomer^a^, Ankor González-Mayorga^e^, Noelia Rodríguez-Díez^a^, Julio Camarero^a,f^, Rodolfo Miranda^a,f^, Elisa López-Dolado^e,g^, Pilar Ocón^h^, María C. Serrano^d^, Lucas Pérez^a,b^ and M. Teresa González^a,*^

^a^ Fundación IMDEA Nanociencia, Calle Faraday 9, 28049 Madrid, Spain

^b^ Departamento de Física de Materiales, Universidad Complutense de Madrid, Plaza de las Ciencias s/n, 28040 Madrid, Spain

^c^ Max Planck Institute for Chemical Physics of Solids, Dresden, Germany

^d^ Instituto de Ciencia de Materiales de Madrid (ICMM), CSIC, Calle Sor Juana Inés de la Cruz 3, 28049 Madrid, Spain

^e^ Hospital Nacional de Parapléjicos, SESCAM, Finca La Peraleda s/n, 45071 Toledo, Spain

^f^ Dept. de Física de la Materia Condensada and Instituto “Nicolás Cabrera”, Universidad Autónoma de Madrid, 28049 Madrid, Spain

^g^ Design and development of biomaterials for neural regeneration, HNP-SESCAM, Associated Unit with CSIC through ICMM, Finca La Peraleda s/n, 45071 Toledo, Spain

^h^ Departamento de Química Física Aplicada, Universidad Autónoma de Madrid, 28049 Madrid, Spain

^‡^ These authors contributed equally

^‖^ Animal Molecular and Cellular Biology group (AMCB), Louvain Institute of Biomolecular Science and Technology (LIBST), Université catholique de Louvain, Place Croix du Sud 5, 1348 Louvain la Neuve, Belgium.

* Corresponding author. Email: teresa.gonzalez@imdea.org


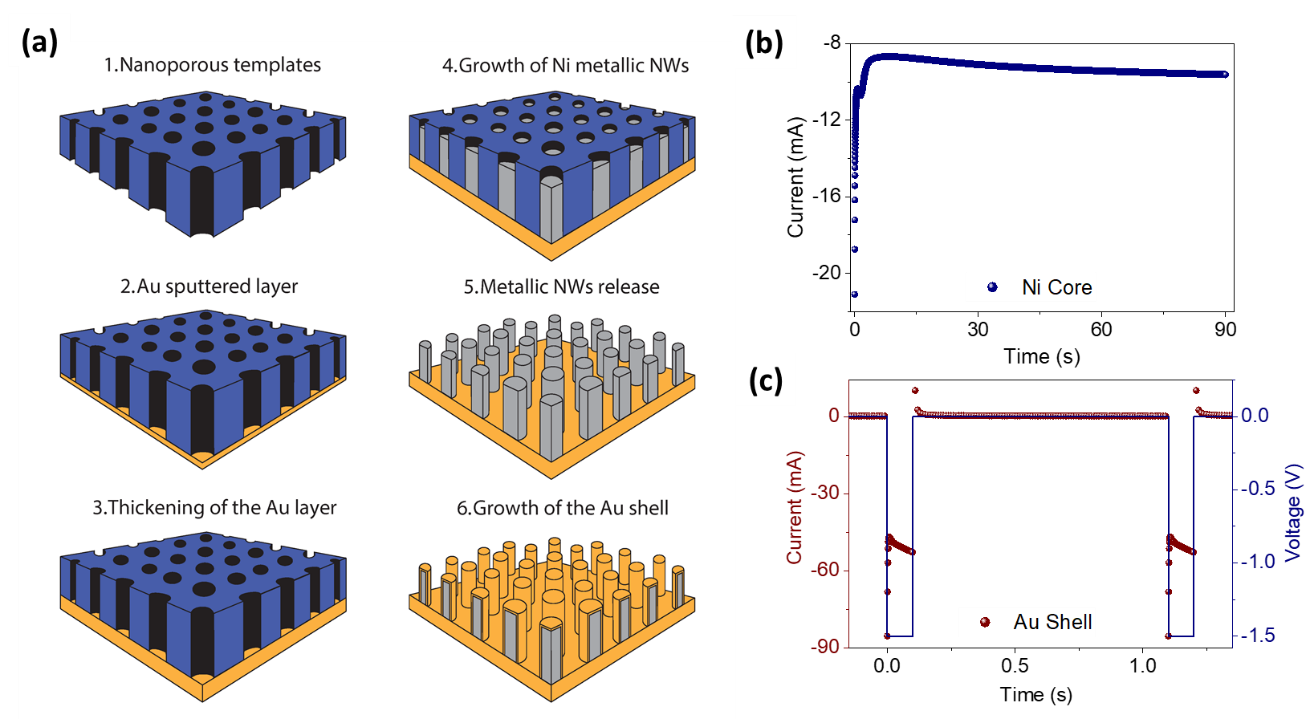


**Figure S1.** Schematics of the protocol followed for the synthesis of the Ni-Au core-shell NW electrodes. The procedure starts from a nanoporous polycarbonate template (1) in which an Au layer is sputtered on the back-side (2). This layer is thickened (3) and the pores filled with Ni (4). Then, the template is removed (5) and the Au shell coating grown (6).


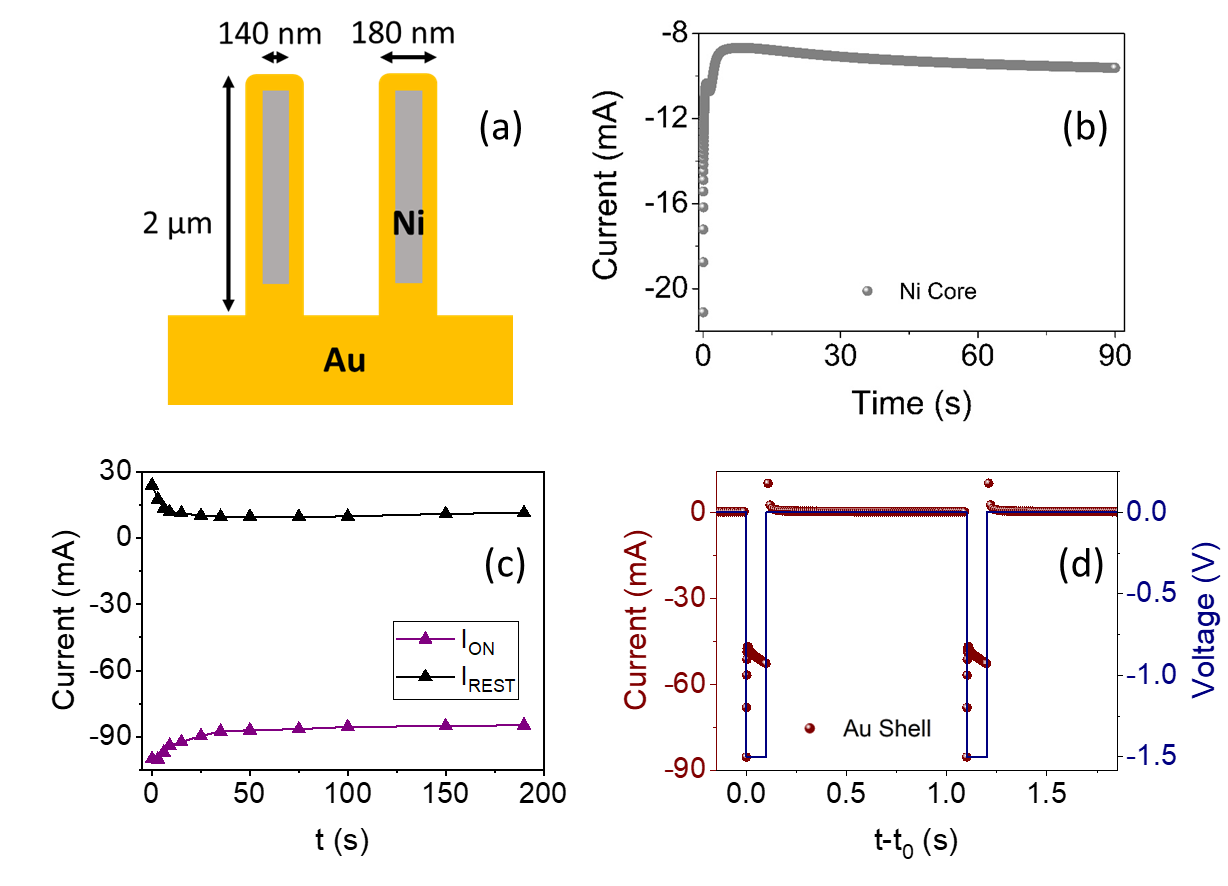


**Figure S2.** a) Schematics of the Ni-Au NW electrodes obtained with PC100 templates. b) Chronoamperometric curve of the Ni NWs electrodeposition inside the pores of the PC membranes. (c) Evolution of the current during the Au shell pulsed electrodeposition. Note that the largest value of the current measured in the pulses corresponding to the V_ON_ and V_REST_ steps is represented. (d) Closer detail of the current measured under the pulses applied when electrodeposition reached the steady state (t_0_ = 120 s).


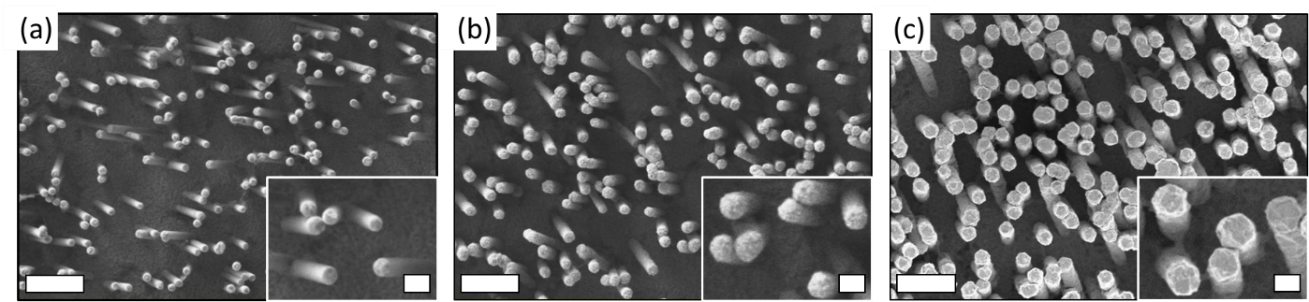


**Figure S3**. (a) Bare Ni NWs with no Au shell. Ni-Au NW electrodes after the Au shell growth applying 175 (b) and 500 pulses (c). An increment in the diameter of the NWs of approximately 40 nm after 175 pulses (thickness of the Au shell of ≈ 20 nm) and 100 nm after 500 pulses (thickness of the Au shell of ≈ 50 nm) was observed. Scale bars: 1 µm (main images) and 200 nm (insets).


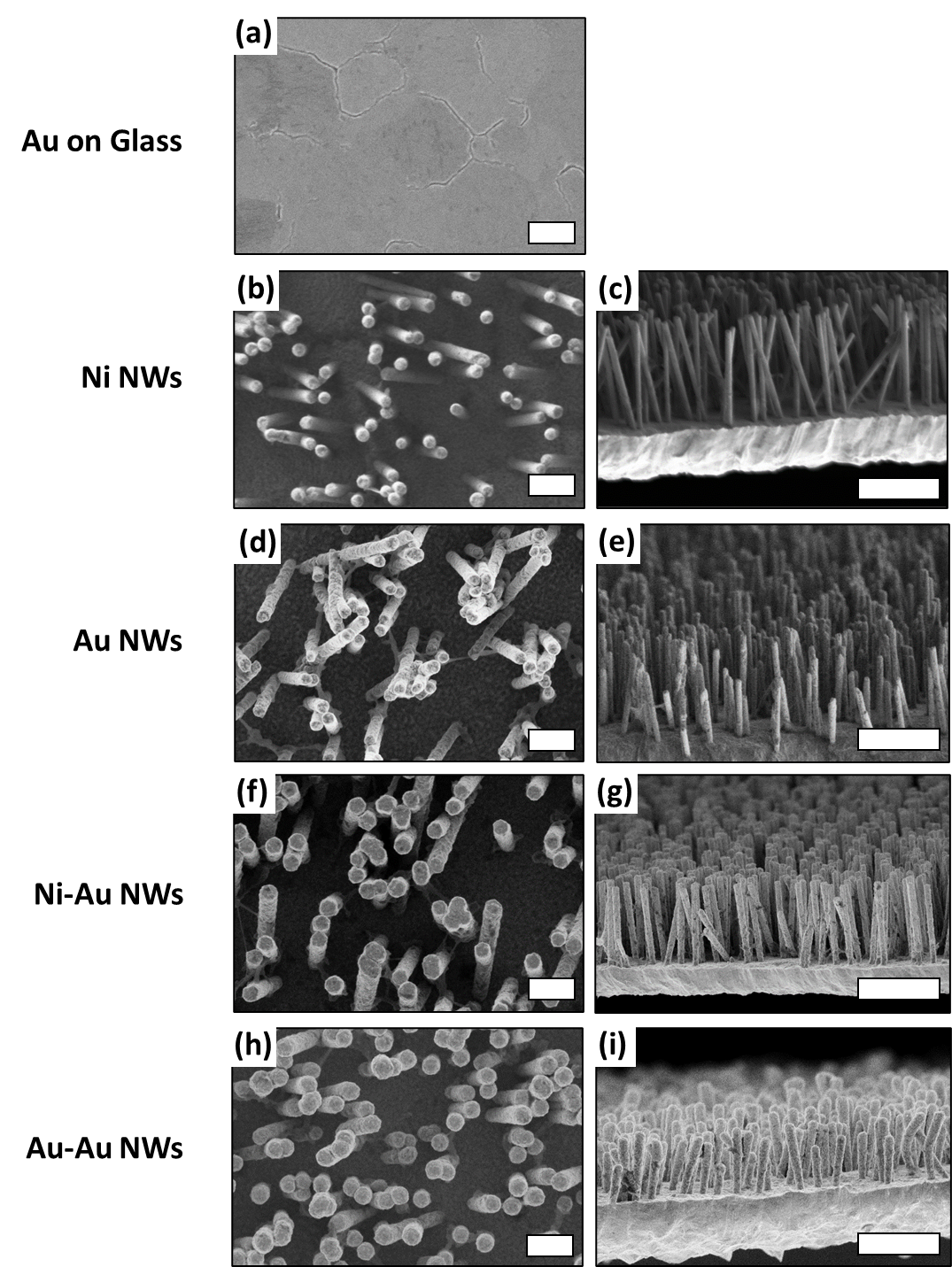


**Figure S4.** (a) FESEM image of an Au on glass substrate (top view). (b-i) FESEM images of Ni NW (b and c), Au NW (d and e), Ni-Au NW (f and g) and Au-Au NW (h and i) electrodes, in top (left column) and cross-section (right column) views. Scale bars: 500 nm (top views) and 2 µm (cross-section views).

**Figure S5.** Representative voltammograms of Ni-Au NW (blue), Au NW (orange) electrodes and Au on glass substrates (black).


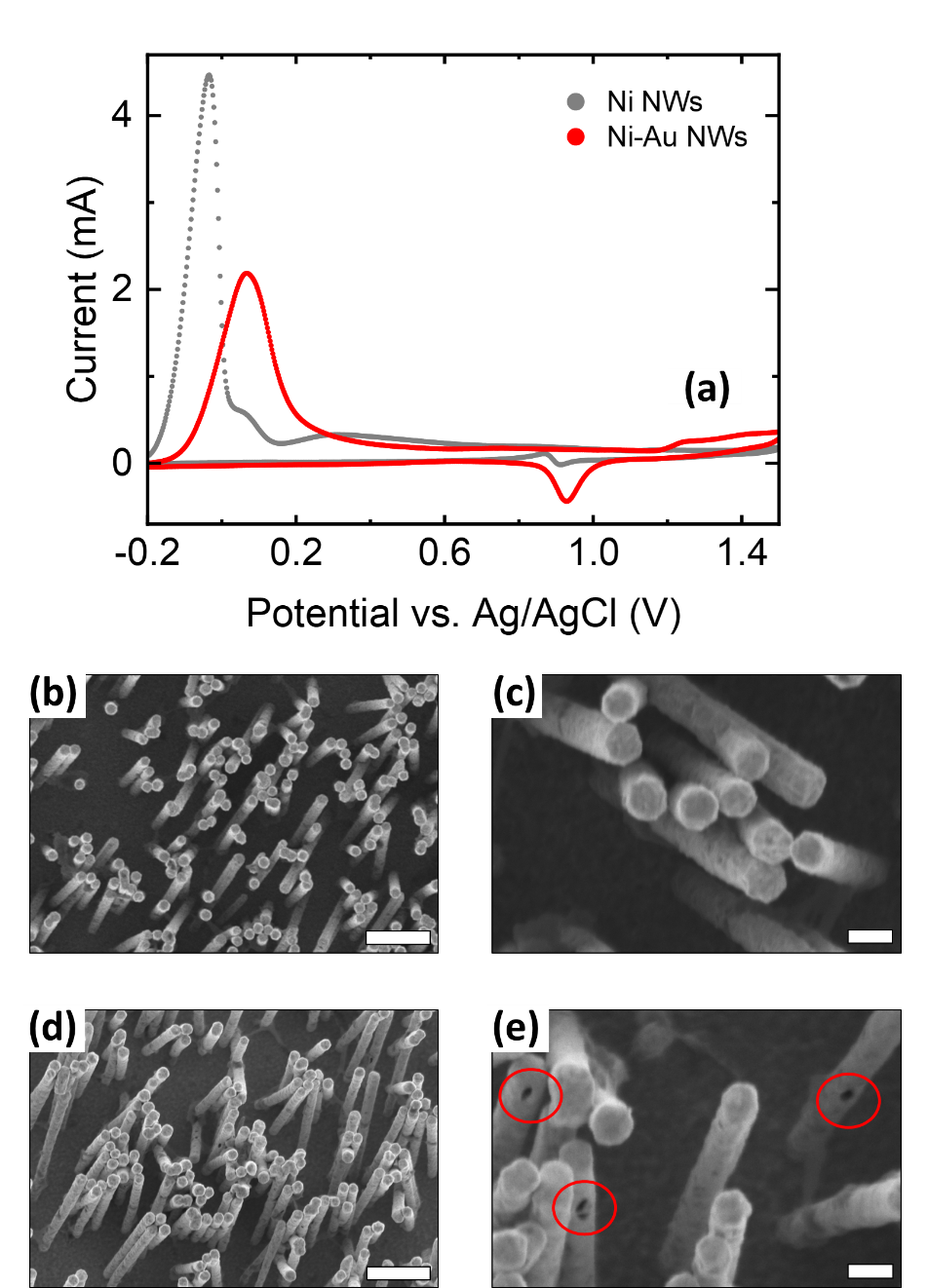


**Figure S6.** – a) Voltammogram of a Ni-Au NW electrode (red) with an incomplete Au shell, evidencing Ni electrochemical activity. The Au shell covering is incomplete due to a short (insufficient) shell electrodeposition time. The voltammogram of a Ni NW (grey) electrode is included as reference. FESEM images of Ni-Au NW electrodes with a complete shell coverage (after 175 cycles of Au shell growth; b,c) and with an incomplete coverage (d,e) evidenced by holes in the Au shell (indicated by red circles). Scale bars: 1 µm (b,d) and 200 nm (c,d).


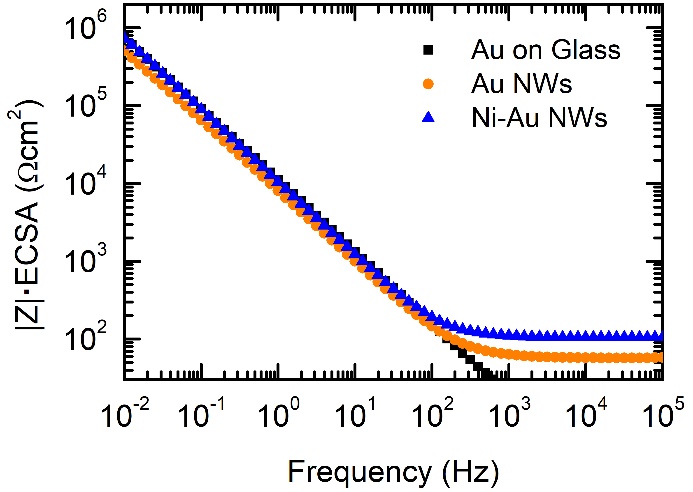


**Figure S7.** |*Z*| *vs* frequency plot scaled by the ECSA of each type of sample obtained from CV measurements. A very good agreement is obtained among them confirming that the obtained ECSA values are indeed the electrochemical active areas.

**Nyquist Plots**

**Bode Plots**


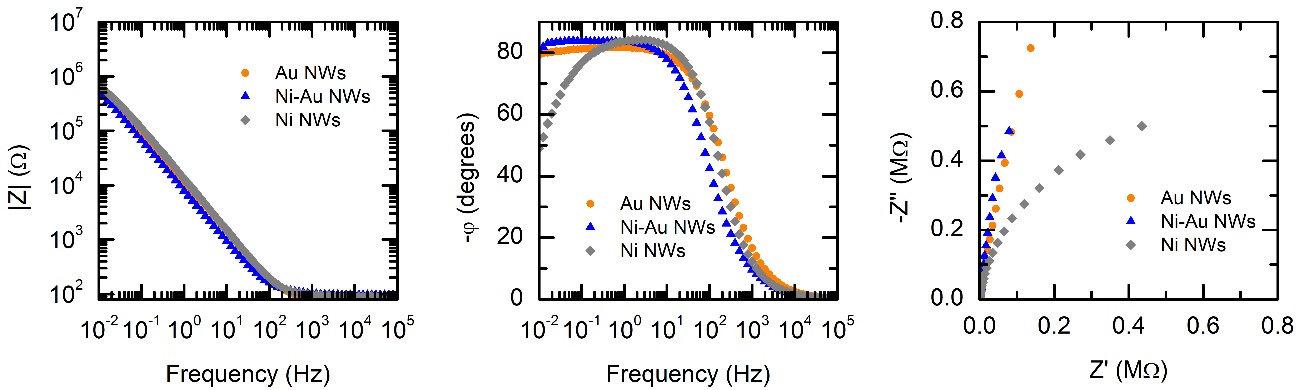


**Figure S8.** Bode and Nyquist plots of the electrodes with different NW composition. The impedance of the Ni-Au NW electrodes is very similar to that of Au NW electrodes in the whole range of frequencies, while, for Ni NW electrodes, -ϕ significantly decreased below 1 Hz.

**Nyquist Plots**

**Bode Plots**


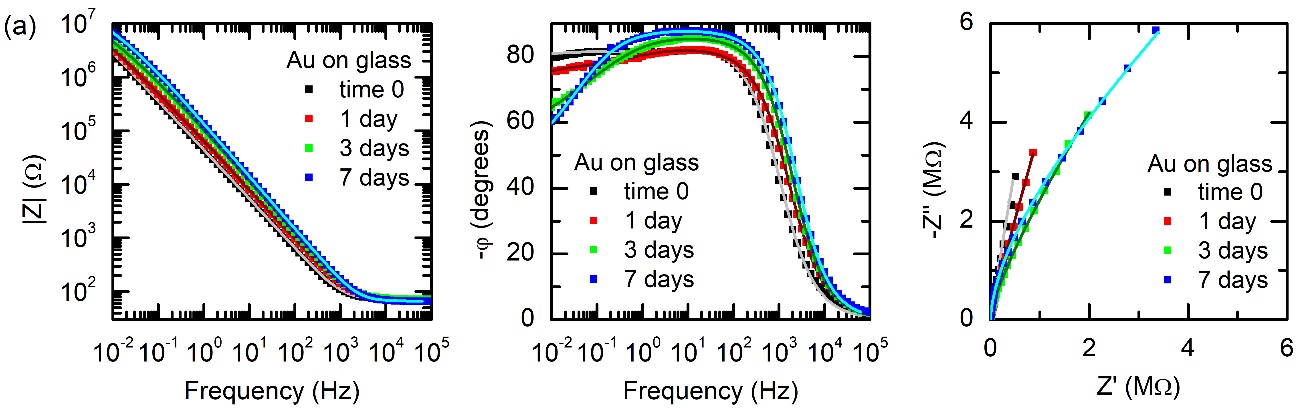

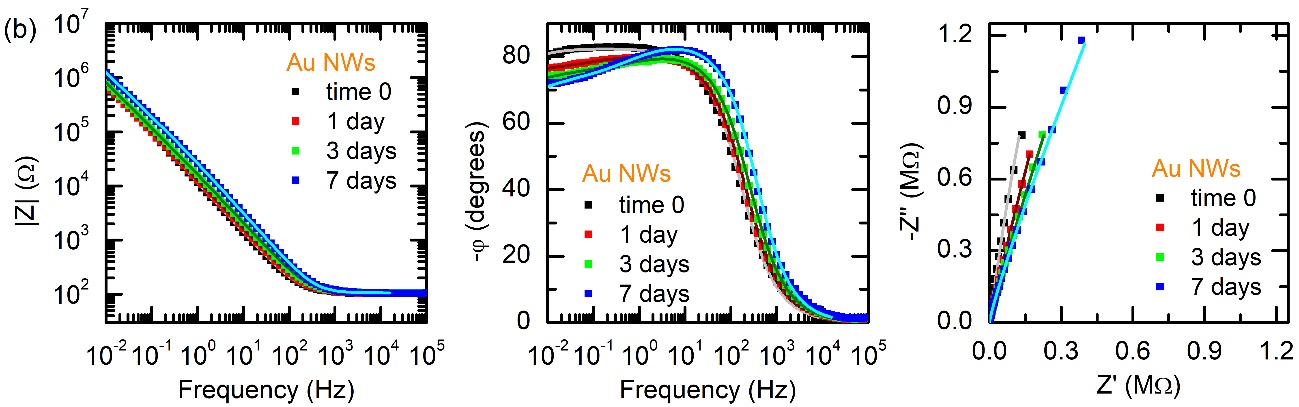

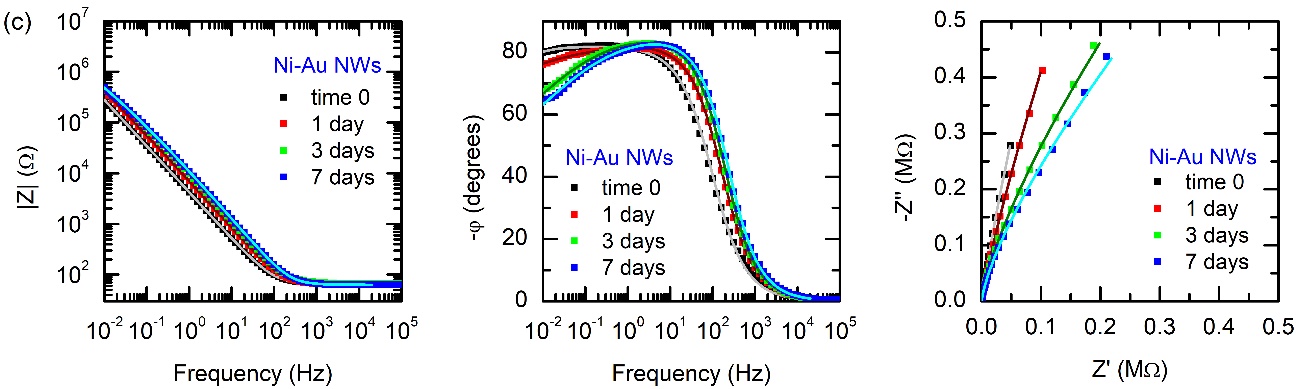

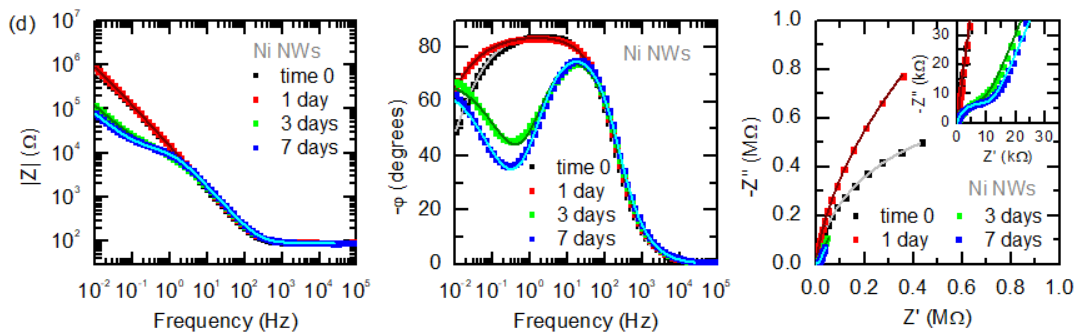


**Figure S9.** Examples of the variation of the Bode and Nyquist plots over a 7-days period for all the electrode types studied. Square points correspond to experimental data, while the continuous lines are the resulting impedance fits of the equivalent electrical circuit (Figure 5a in the main text). At time cero, CPEw was not included in the model for Au NWs and Ni-Au NWs. The exact evolution of the plots was slightly different for each individual sample of the same type of electrode.


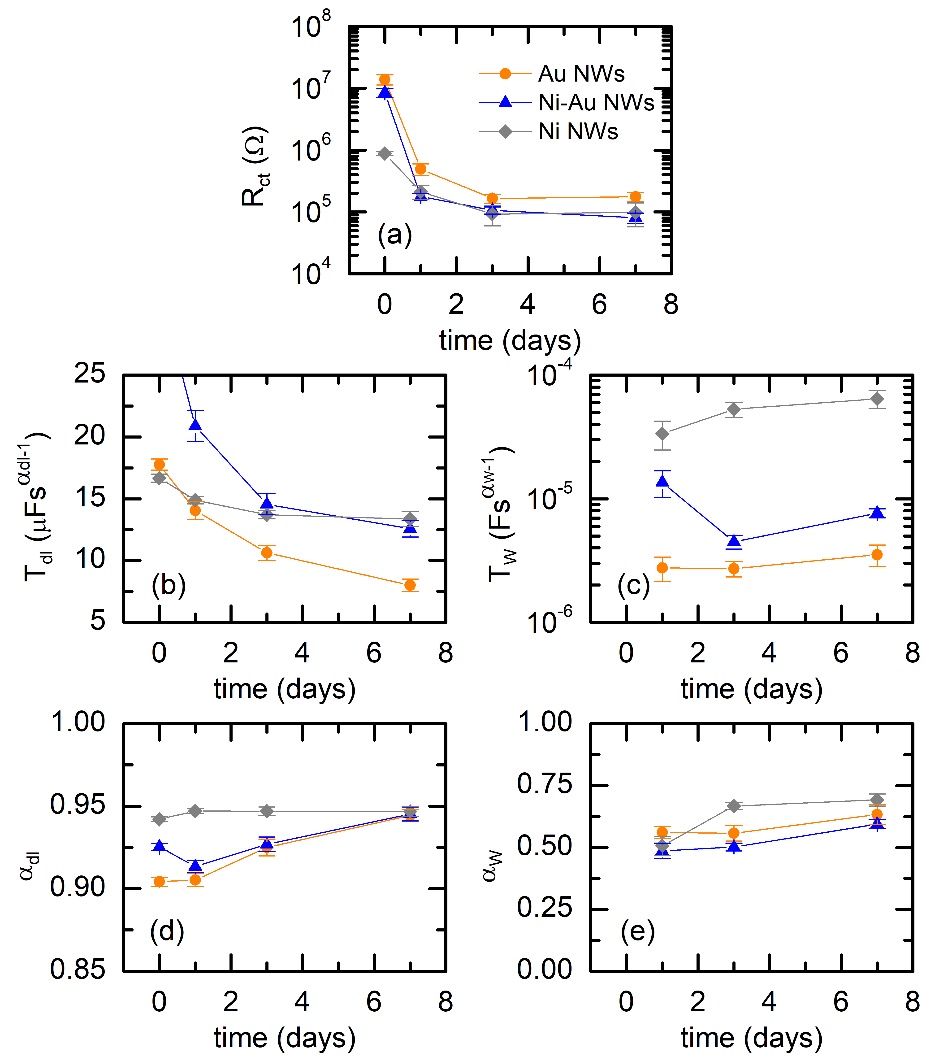


**Figure S10.** Variation along time of the resulting fitting parameters from the equivalent electrical circuit (corresponding to Figure 5a in the main manuscript) for electrodes with different NW composition. CPE_dl_ is a constant phase element that accounts for a non-purely capacitive double-layer capacitance with some frequency dispersion. CPE_W_ accounts for the capacitive part of the diffusion or mass transfer component. α is a dimensionless phase shift parameter with values between 1 and 0, so that when α=1 the CPE is a pure capacitor with capacitance T, when α=0, the CPE is a resistor with resistance 1/T and when α=0.5, a pure Warburg diffusion element. R_ct_ is the charge transfer resistance. The variation trend of T_dl_ of Ni NW electrodes is different from that of Au and Ni-Au NW electrodes. Also, the α_dl_ value remained higher for Ni NW electrodes at the beginning, with little variation over time.


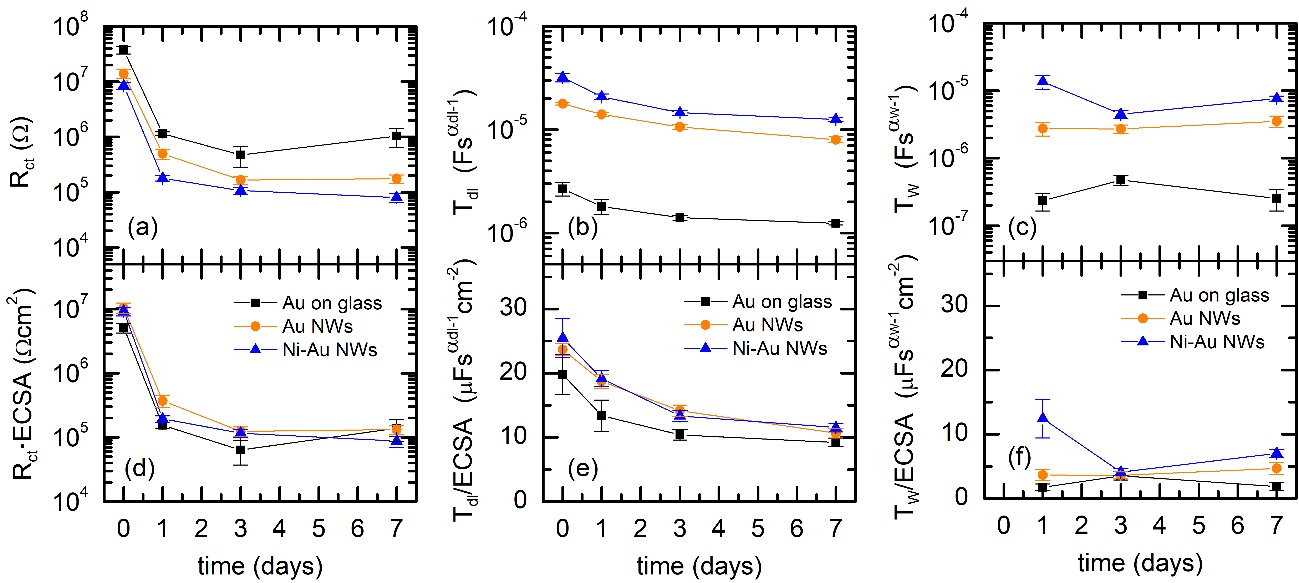


**Figure S11**. R and T parameters of the equivalent electrical circuit (corresponding to Figure 5a in the main manuscript) before (a-c) and after (d-f) scaling with the ECSA values obtained from the CV measurements for Au on glass, and Au NW and Ni-Au NW electrodes (all samples with the same geometric surface area of 0.13 cm^2^. T_dl_/ECSA values agree very well for the four samples investigated. T_W_/ECSA values remained fairly stable in the studied time period for all the samples.
